# Cardiac involvement in female elite athletes with carrier status of Duchenne muscular dystrophy

**DOI:** 10.3389/fcvm.2025.1606994

**Published:** 2025-07-07

**Authors:** Simon Wernhart, Tom Kastner, Martin Halle, Stephan Mueller, Veronika Schmid, Cihan Akbulut, Cordula M. Wolf, Christian Meierhofer, Teresa Trenkwalder, Isabel Diebold, Christopher Herzog, Richard Brill, Mark J. Haykowsky, Stephen Foulkes, Bernd Wolfarth, Eimo Martens, Dominik S. Westphal

**Affiliations:** ^1^ Department for Preventive Sports Medicine and Sports Cardiology, TUM School of Medicine and Health, TUM University Hospital, Technical University Munich (TUM), Munich, Germany; ^2^ DZHK (German Centre for Cardiovascular Research), Munich Heart Alliance, Munich, Germany; ^3^ Department of Sports Medicine, Charité-Universitätsmedizin Berlin and Humboldt-Universität zu Berlin, Berlin, Germany; ^4^ Institute for Applied Training Science, Leipzig University, Leipzig, Germany; ^5^ Department of Congenital Heart Defects and Pediatric Cardiology, German Heart Center Munich, TUM School of Medicine and Health, TUM University Hospital, Technical University Munich (TUM), Munich, Germany; ^6^ Department of Cardiology, German Heart Centre Munich, TUM School of Medicine and Health, TUM University Hospital, Technical University Munich (TUM), Munich, Germany; ^7^ MGZ - Medizinisch Genetisches Zentrum, Munich, Germany; ^8^ Radiologie München im Rotkreuzklinikum, Munich, Germany; ^9^ Clinic and Policlinic of Radiology, University Hospital Halle (Saale), Halle (Saale), Germany; ^10^ Integrated Cardiovascular and Exercise Physiology and Rehabilitation (iCARE) Laboratory, College of Health Sciences, University of Alberta, Edmonton, AB, Canada; ^11^ Hochgebirgsklinik Davos, Davos, Switzerland; ^12^ Heart, Exercise and Research Trials Lab, St. Vincent’s Institute of Medical Research, Melbourne, VIC, Australia; ^13^ Department of Internal Medicine I, TUM School of Medicine and Health, Technical University Munich (TUM), Munich, Germany; ^14^ Europäische Referenznetzwerke – Europäische Kommission, European Reference Network Guard Heart, European Union, Munich, Germany; ^15^ Institute of Human Genetics, TUM School of Medicine and Health, TUM University Hospital, Technical University Munich (TUM), Munich, Germany

**Keywords:** cardiomyopathy, exercise, muscular dystrophy, sports cardiology, preparticipation screening

## Abstract

Duchenne muscular dystrophy is a muscle-wasting, progressive, X-linked inherited disease in young male individuals, who—aside from peripheral muscular impairment—may also suffer from severe cardiac complications. In women who are muscular dystrophy carriers (MDCs), muscular symptoms and cardiac complications are less severe or even absent. While male individuals with muscular dystrophy are not usually able to perform strenuous exercise, women who are MDCs can exercise at mild, or even high, intensity. However, the impact of participating in elite sports, particularly endurance sports with high cardiopulmonary exercise strain, on female athletes who are MDCs is uncertain. Herein, we describe two rare cases of female elite athletes who are MDCs who participated in endurance sports. We describe their clinical presentation, kinetics of cardiac biomarkers and peripheral muscle enzymes during acute exercise, and cardiac manifestations in the context of sports eligibility, including an interdisciplinary shared decision-making approach to whether to continue participating in sports. This approach focuses on pathophysiology and genetics in dystrophinopathies, with a particular focus on genetic carrier status. While the primary concern is risk stratification for sudden cardiac death and its prevention, the potential risk of early onset of myocardial dysfunction or even heart failure also needs to be considered in MDCs. To optimize exercise recommendations, these complex and rare cases of athletes require an interdisciplinary approach, including experts in sports cardiology, sports medicine, radiology, and genetics, and should be included in a long-term international sports cardiology registry.

## Graphical Abstract

**Figure:**
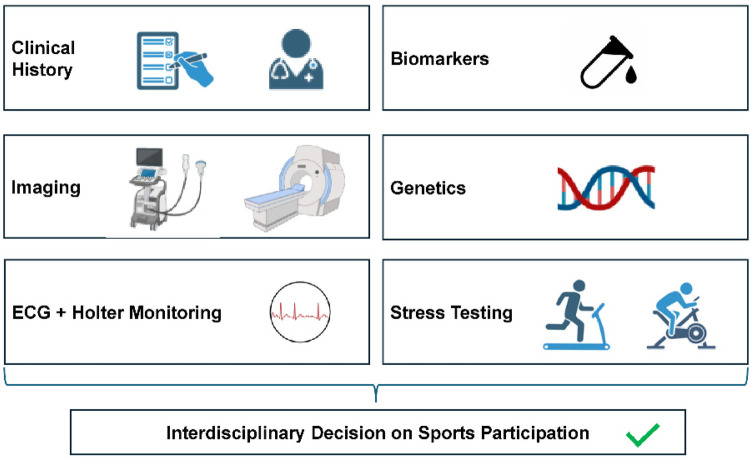
Components of preparticipation screening in muscular dystrophy carriers (MDCs). Screening of patients with MDC should include several components, starting with a meticulous clinical and family history. Electrocardiography and Holter monitoring (if clinically indicated) should complete baseline examination. Imaging (transthoracic echocardiography and cardiac magnetic resonance imaging) should be applied when clinically indicated. Biomarkers should include creatine kinase and glutamate-pyruvate transaminase levels and may contain troponin I and T, especially if suspicion for cardiac involvement is raised. In the future, genetic testing may yield additional information depending on the type of mutation. Clearance for sports participation should require exertional exercise testing without relevant arrhythmias. Images created with biorender.com.

## 1 Introduction

Duchenne muscular dystrophy (DMD) is a muscle-wasting, progressive, X-linked inherited disease affecting 1 in 5,000 boys and is caused by dystrophin protein deficiency (1). Early impairment of mobility and non-achievement of developmental milestones often lead to the diagnosis within the first 5 years of life. Muscular dystrophy carriers (MDCs, i.e., women carrying a pathogenic DMD variant on one X chromosome) are usually asymptomatic, but, in rare cases, they can develop clinical symptoms. Approximately 3%–19% of MDCs have skeletal muscle symptoms, and 7.3%–16.7% develop dilated cardiomyopathy (2), the latter having an impact on prognosis (3).

Recommendations for exercise and training, considering frequency, duration, volume, and, especially, intensity, have not been postulated for MDCs. Furthermore, to our knowledge, thus far, there have been no reports published on the combination of being an MDC, cardiac involvement, and elite sports. We report two young MDC cases without persistent symptoms but with cardiac involvement, one of whom is an elite athlete participating in long-distance cross-country skiing (24 years of age), the other a recreational athlete engaging in regular high-intensity endurance sports (ice skating, 19 years of age). We critically discuss the underlying pathophysiology, clinical presentation, and diagnostics in the context of sports eligibility and describe the need for a multidisciplinary approach and a shared decision-making approach between the expert consensus and the athlete. Therefore, these two cases are examples of how to address the problem of sports eligibility in athletes with a genetic carrier status associated with potential cardiac involvement.

## 2 Genetics and pathophysiology of dystrophinopathies

DMD is caused by pathogenic variants in the DMD gene, which encodes dystrophin, that prevent the production of the muscle isoform of dystrophin. Pathogenic variants in the DMD gene can also cause Becker muscular dystrophy (BMD), which is a milder disease with a later onset and a slower progression than DMD. In DMD, pathogenic out-of-frame variants or non-sense variants lead to non-functional and unstable dystrophin. By contrast, BMD is caused by in-frame deletions or duplications that result in a protein that is partially functional. Deletions are the most common type of pathogenic variants in the DMD gene (found in approximately 60%–70% of cases), while duplications are less common (approximately 10% of DMD cases) (4).

There is limited evidence of a possible genotype–phenotype correlation in MDCs. However, there is some evidence that MDCs carrying a deletion in the DMD gene may be asymptomatic more frequently than those with duplications (5, 6). A functional dystrophin protein deficiency impairs the anchoring function of the dystrophin–glycoprotein complex involved in cell signaling and preservation of membrane stability during (especially eccentric) muscle contraction (7). As such, impaired dystrophin is especially vulnerable to eccentric stimuli, inducing disruption of the sarcolemma, which distorts calcium homeostasis and leads to increased proteolysis (8, 9). The prognosis for MDCs largely depends on whether there is unfavorable X-inactivation. If the mutated X chromosome is preferentially expressed, symptoms such as muscle weakness, cardiomyopathy, or elevated creatine kinase (CK) levels can occur (10–12).

## 3 Cardiac involvement in dystrophinopathies

Cardiac muscle involvement may occur in MDCs (13, 14), but the prevalence varies among reports, with some demonstrating a prevalence of up to 40% (10, 15, 16). In a study of 129 MDCs (85 with Duchenne and 44 with Becker dystrophy, aged 18–60 years) with 37 mutations, no genotype–phenotype association was found (15). In total, 22% of the women had symptoms, predominantly muscle weakness (17%), 18% displayed left ventricular dilation, and 8% were diagnosed with dilated cardiomyopathy. In a prospective cohort study, 48% of 77 genetically confirmed MDCs [mean age of 41.3 years; mean left ventricular ejection fraction (LVEF) of 59.2%] showed diffuse late gadolinium enhancement (LGE) on cardiac magnetic resonance (CMR) imaging as a sign of cardiac involvement without manifest limitations in cardiopulmonary exercise testing (CPET) or clinical symptoms, with only four patients terminating the exercise due to musculoskeletal symptoms (17). The percentage of LGE was significantly higher among the MDCs compared to the non-carriers (48% vs. 4.5%). Moreover, 25% of the carriers displayed ventricular ectopy during recovery from exercise, while the MDCs who showed LGE on imaging presented with higher levels of CK in the absence of clinical or CPET evidence of peripheral muscular limitations (17). Thus, disproportionately higher serum CK levels in athletes in combination with non-ischemic scars on CMR should prompt physicians to widen their differential diagnosis from residuals of myocarditis to systemic disease, in spite of a high exercise capacity and a lack of skeletal muscle symptoms (18).

## 4 Case #1: 24-year-old female elite athlete participating in cross-country skiing

**Figure 1:**
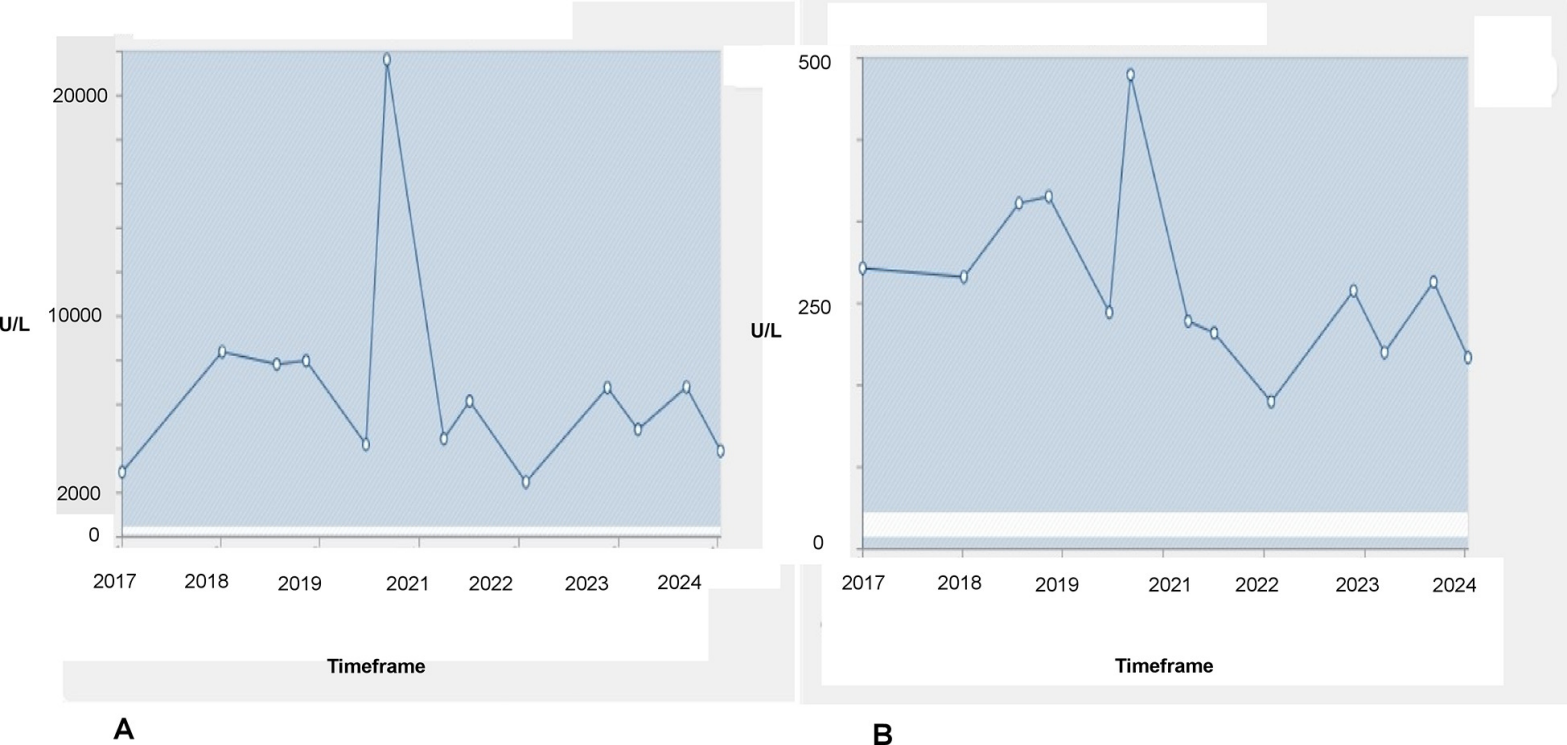
Changes in CK **(A)** and GPT **(B)** levels from 2017 to 2024. Reference values are 24–350 U/L for CK and 10–35 U/L for GPT. The horizontal white lines represent the reference values for the parameters.

At the age of 17 years, the athlete reported to us for the first time for a routine preparticipation screening as a cross-country skier. Previously, at the age of 6 years, an exaggerated increase in CK level (>19,000 U/L) was recorded following intensive training that had led to further diagnostics, including genetic testing for muscular dystrophy. This genetic testing found a heterozygous in-frame duplication of exons 9–44 in the DMD gene. As the athlete was asymptomatic, eligibility for competitive sport was confirmed at that time. Since then, CK and glutamate-pyruvate transaminase (GPT) levels were repeatedly measured during routine sports cardiological consultations, which revealed considerable deviations (see Figure 1). Exercise volume was kept steady over the years at an estimated weekly duration of 20 h, with the athlete engaging in predominantly high-intensity endurance activities.

**Figure 2:**
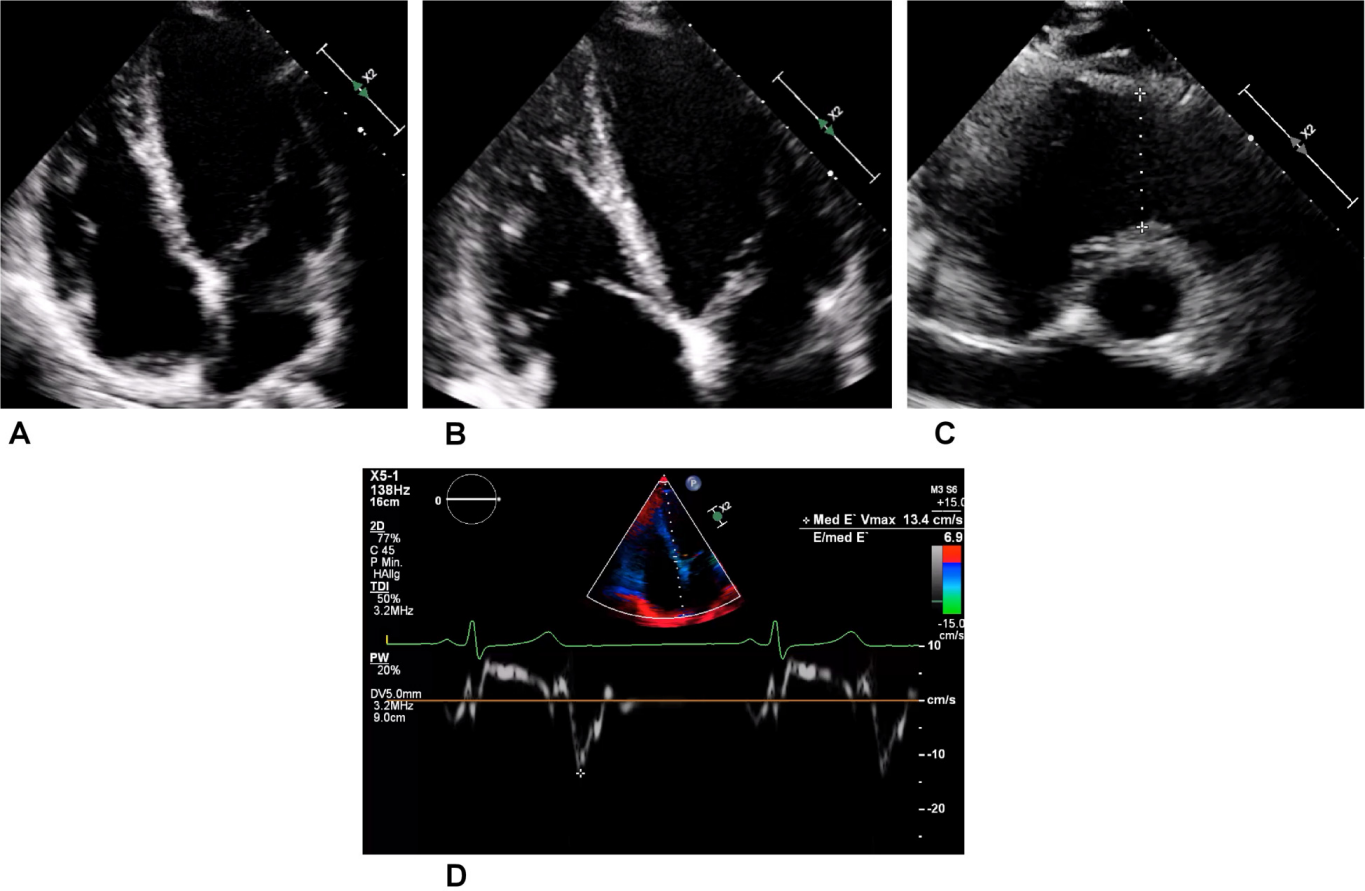
Transthoracic echocardiography of the asymptomatic athlete. **(A)** Regular four-chamber view. **(B)** Right dominant four-chamber view showing a trabeculated free wall in the right ventricle. **(C)** Slight dilatation of the right ventricular outflow tract (a distance of 34 mm is represented by the dotted line) in this normally sized young woman. **(D)** Medial tissue Doppler imaging revealing regular velocity.

During this period, the athlete, who was still asymptomatic, took part in a training study in which troponin T levels were measured. These showed intermittent minor increases up to 30 ng/L (reference <14 ng/L) on non-exercising days. Notably, the athlete’s troponin I level was permanently within normal range, and the electrocardiogram (ECG) showed sinus rhythm without ST-segment alteration or T-wave inversions. Subsequent transthoracic echocardiography revealed a normal biventricular ejection fraction and diastolic function. Only the lateral aspect of the right ventricular (RV) free wall showed some hypertrabeculation, but the RV did not display apical aneurysms or hypokinesia (Figures 2A–D).

**Figure 3:**
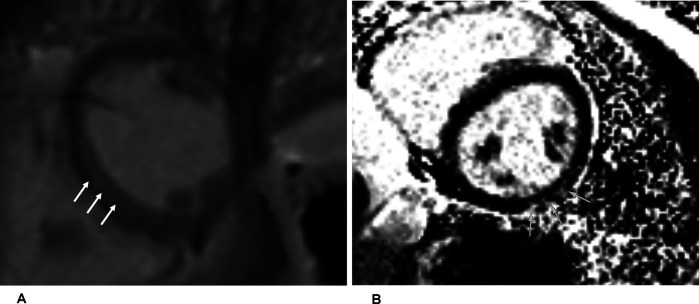
Magnetic resonance imaging of the heart (short-axis views). LGE is displayed in the epicardial midventricular **(A)** and patchy inferolateral **(B)** areas. The arrows refer to the LGE.

For verification, CMR imaging was conducted, which revealed LGE in the epicardial midventricular wall (length of 3 cm) (Figure 3A) and patchy inferolateral areas in the left ventricle (LV) (Figure 3B). No clinical history of infection had been reported. Equivalent radiological examinations were repeated thrice within the following 3 years, and these did not show any morphological progression of the LGE.

**Figure 4:**
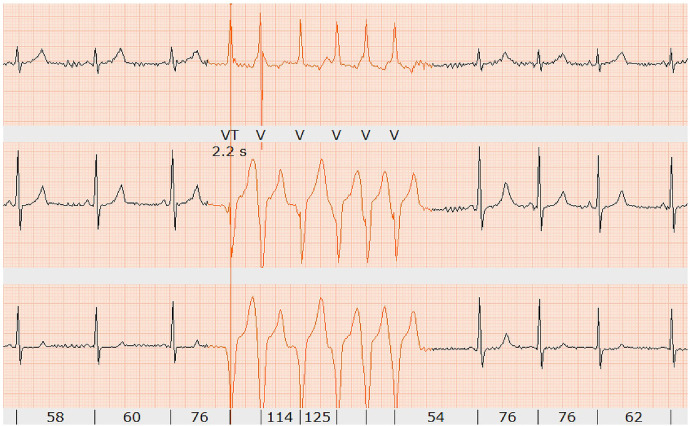
Holter monitoring showing short non-sustained ventricular tachycardia (six beats in red) at rest (confirmed by two independent electrophysiologists).

During the placement of the venous cannula for the injection of the CMR contrast agent, the athlete fainted and showed asystole for 18 s, which was captured by Holter monitoring. No previous syncope or palpitations had been reported. During the regular follow-up period, Holter monitoring was added, which displayed short, non-sustained ventricular tachycardia during rest (Figure 4). As this was three-lead Holter monitoring, the origin of the tachycardia could not be located.

Moreover, for this athlete, we conducted an exercise challenge using exertional bicycle ergometry. No arrhythmia was induced until maximal exhaustion and a maximal performance of 230 W (peak heart rate 189 /min, 107% of predicted). The athlete’s resting CK level of 4,806 U/L increased to a post-exercise level of 5,662 U/L (reference <350 U/L). Troponin I levels were negative (below the 99th percentile) on both measurements, while troponin T measurements were not available. The athlete’s N-terminal prohormone of brain natriuretic peptide (NTproBNP) levels were within the normal range.

The case was discussed by an interdisciplinary panel of sports cardiologists, electrophysiologists, pediatric cardiologists, radiologists, and genetic experts. Taken together, the transient and disproportionate elevation of CK (attributed to peripheral muscular strain and underlying dystrophinopathy) (18) and increases during the exercise challenge confirmed peripheral muscular membrane instability in this athlete, who is an MDC. An elevation in the CK level >20.000 U/L following endurance training is not expected in a healthy individual and should raise suspicion for underlying muscular disease. The asystole detected in the CMR imaging was characterized as a neuro-cardiogenic syncope due to venous puncture. However, overall, the minor repetitive troponin T elevations and myocardial fibrosis in the CMR imaging, in combination with non-sustained ventricular tachycardia, were judged to be pathological, and cardiac involvement related to being MDC was considered plausible. Following a shared decision discussion, the athlete withdrew from competitive endurance sports participation and focused on recreational, low-intensity exercise.

## 5 Case #2: 19-year-old female recreational athlete participating in ice skating

A diagnosis of being an MDC was given to a female recreational athlete in ice skating (weekly exercise training duration of 10 h) at the age of 6 years (deletion of exons 49–50 in the dystrophin gene) due to repetitive bouts of muscle pain and increases in CK level above 5,000 U/L despite limiting exercise. Despite these intermittent muscle symptoms, participation in recreational sports was continued. However, starting at the age of 15 years, she had repeatedly reported to the emergency hospital unit due to thoracic pain. Electrocardiographic findings, as well as troponin T and NTproBNP levels, were always within normal ranges, while the athlete’s troponin I level was slightly elevated once at the age of 19 (peak 119 ng/L, reference <35 ng/L), with transthoracic echocardiography revealing preserved biventricular function (65% of LV ejection fraction, biplanar LVEF) without wall motion abnormalities and no signs of diastolic impairment at this time. Due to transient biomarker elevation, CMR imaging was performed at the age of 19 years. This revealed no acute injury or signs such as myocardial edema, but a positive LGE was observed at the inferolateral aspect of the left ventricle. Due to ongoing thoracic pain and exclusion of coronary anomalies on initial CMR imaging, a diagnosis of subacute myocarditis was made despite a lack of edema, and cessation of sports participation was recommended for at least 3 months according to current guidelines (19, 20).

**Figure 5:**
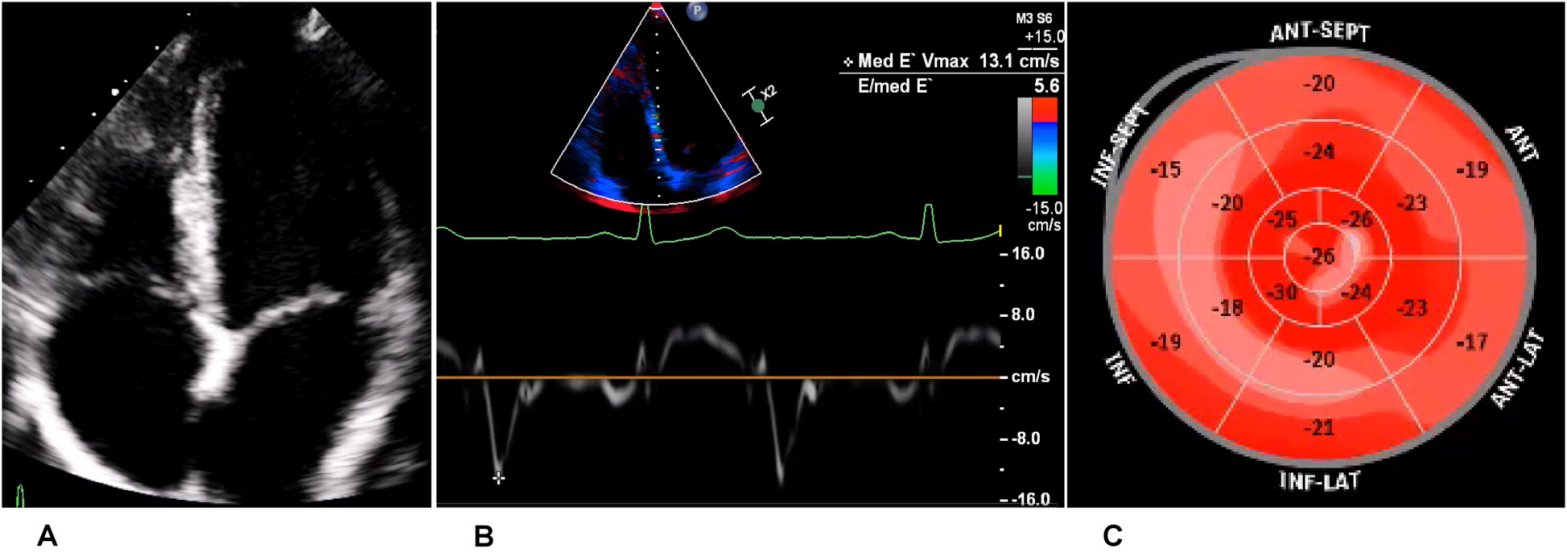
Echocardiography of the recreational athlete (ice skating). **(A)** Four-chamber view on transthoracic echocardiography showing no signs of structural heart disease. **(B)** Medial tissue Doppler imaging demonstrating velocity within the normal range. **(C)** Bullseye display of the distribution of longitudinal strain, with more positive values depicting worse strain. This was demonstrated in the inferior, inferolateral, and inferoseptal regions, corresponding to the LGE on magnetic resonance imaging.

**Figure 6:**
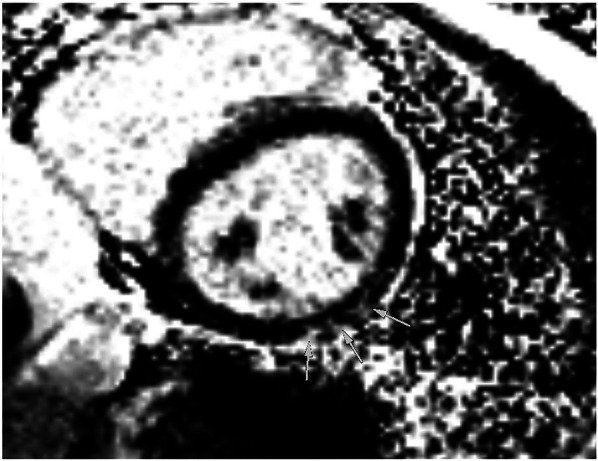
Magnetic resonance imaging at follow-up showing inferolateral late gadolinium enhancement illustrated by arrows in a short-axis view, corresponding to regions of reduced strain in echocardiography.

The athlete was lost to follow-up for 1 year (between 19 and 20 years of age), but another echocardiogram, performed at the age of 20 years, showed a slight decline of biplanar LVEF to 58%. However, the athlete had normal diastolic function and no signs of structural heart disease (Figures 5A,B). Strain analysis showed that the athlete’s global longitudinal strain was within the normal range (−22.3%), with reduced values in the inferolateral, inferior, and inferoseptal areas (Figure 5C). Cardiac biomarkers (troponin and NTproBNP) were within the normal range. CMR imaging was performed again and showed reproducible, consistent LGE of the inferolateral wall without edema (Figure 6). However, LVEF had declined from 69% to 53% within 1 year (from age 19 to 20 years). The athlete had, of her own accord, resumed recreational sport activity between 19 and 20 years of age.

As there was no sign of acute edema and negative cardiac biomarkers, exertional CPET was performed at the age of 20 years and showed no signs of arrhythmia at a good fitness level (3.0 W/kg, VO_2_peak 42.4 ml/kg/min, 116% of predicted), which was compatible with normal Holter monitoring. We observed no signs of circulatory limitations (peak oxygen pulse of 12.5 ml/beat, 133% of predicted, no plateauing; increase in end-tidal carbon dioxide of 11 mmHg during exercise). The regular increase in end-tidal carbon dioxide during exercise suggested adequate alveolar perfusion (together with preserved minute ventilation/ oxygen uptake) and diaphragmatic competency. However, retention of carbon dioxide during exercise could be an early sign of exercise-induced diaphragmatic insufficiency in muscular disease. Gas exchange was not disturbed (alveolar-arterial gradient at rest 22 and 18 mmHg at peak exercise), and there were no signs of ventilatory limitations during exercise (peak minute ventilation of 65.9 L, 110% of predicted; breathing reserve of 30%, slope of minute ventilation to carbon dioxide production of 29.8). During flow-volume registration, a slight decrease in inspiratory capacity (from 1.88 to 1.73 L) and an increase in end-expiratory lung volume (from 1.59 to 1.68 L) were observed but were not considered clinically relevant. However, given the fact that respiratory failure can occur during the course of the disease, follow-up CPET testing was recommended after 1 year. Unfortunately, we did not have CPET data from when the athlete was 19 years old or younger to correlate the exercise data with the athlete’s resting LVEF on echocardiogram and CMR imaging.

The data and clinical presentation were discussed by the interdisciplinary panel and were interpreted to be cardiac involvement in an MDC and a potential concomitant minor myocardial reaction to infection, rather than myocarditis. As maximal CPET was normal, clearance for recreational sports was given. However, as ice skating requires changes in concentric and eccentric muscle work and sudden bouts of higher intensity [with potential detrimental effects in dystrophinopathies (21, 22)], we counseled the athlete to switch to low-intensity, endurance exercise activities and short isometric resistance training, such as wall-sit exercises or planks. As the athlete’s repeated visits to the emergency unit did not reveal symptoms of acute pathology, reassurance and psychological support were offered. Given the fact that the athlete’s CK levels did fluctuate, non-cardiac thoraco-muscular involvement was viewed as possible but did not prompt exclusion from routine sports participation. We recommended a cardiological follow-up 6 months after the follow-up CMR imaging with resting and stress echocardiography, including strain analysis and CPET. Exercise testing is a valuable tool to assess cardiopulmonary function during exercise, but it can also be used to assess exercise-induced arrhythmias in individuals with potential cardiac involvement in muscular disease. Drug therapy was not initiated as the ejection fraction was above 50%, but this may need to be considered if there is a further decline in the athlete’s resting and stress-induced LVEF. In summary, in this case, cardiac involvement in muscular disease cannot be excluded, but in the absence of arrhythmias and manifest cardiopulmonary limitations, low-intensity recreational sports should be encouraged, provided cardiological follow-up examinations are conducted. It must be stated, however, that the differential diagnosis and optimal workup of athletes with suspected myocarditis remain unknown and require further prospective investigation (23, 24).

## 6 Discussion

Herein, we describe two rare cases of MDC athletes with potential cardiac involvement who participated in high-intensity sports at recreational and elite levels, respectively. In both cases, an interdisciplinary discussion led to exercise prescriptions that were based on expert rather than guideline recommendations. As no exercise studies on MDCs with cardiac involvement exist, these cases stress the importance of interdisciplinary cooperation. In the elite athlete, syncope (although most likely triggered by venous puncture), non-sustained ventricular tachycardia, and the morphological CMR imaging changes led to a recommendation to cease participation in competitive sports. In contrast, the recreational athlete was reassured that she could continue regular exercise, despite an obvious fear of life-threatening events and repetitive emergency unit consultations. We recommended that both athletes continue with regular sports cardiological follow-ups, low-intensity isometric resistance training, and endurance exercise below each individual’s anaerobic threshold. However, it must be acknowledged that these recommendations were not based on evidence from human studies but rather on a rational cardiological approach. There is no solid data on the effects of volume expansion (e.g., pregnancy or valvular heart disease) in MDCs, which may help delineate the effect of pressure and volume load on the heart and derive exercise recommendations. We hypothesize that a higher intensity could lead to more catecholaminergic stimulation, which may be a trigger for arrhythmias. However, it must be considered that low-intensity exercise also exerts cardiac stress and may, in the end, be proarrhythmogenic. We further hypothesize that the arrhythmogenic effect could include parameters such as loading conditions and exercise intensity, duration, and density. In general, health guidelines should be met by avoiding excessively long exposure to exercise and strenuous activities that increase one’s sympathetic tone. Clearly, more long-term (mechanistic and clinical) studies are needed to tailor safe exercise prescriptions in these rare cases.

As there is evidence from mouse studies that eccentric muscle work can be detrimental in patients with dystrophinopathies, we suggest low-intensity isometric resistance training without net changes in muscle length (22). However, the cellular effects of exercise on MDCs have not been studied; therefore, analogies have to be made from DMD models of the so-called “mdx mouse” with an inherent non-sense variant in exon 23 (25). Endurance exercise studies in this mouse model show conflicting results on the peripheral and cardiac muscles due to the large divergence of protocols in terms of exercise intensity, duration, and frequency (21). In humans, both inspiratory and expiratory muscle training have shown improvements in respiratory endurance (26), strength (27), and vital capacity (28) in patients with DMD.

There are equivocal data in DMD and non-existent data from among MDCs on the effect of different modes and durations of exercise on myocardial integrity. In patients with DMD, acute bouts of exercise, depending on the intensity and duration, can result in significantly higher levels of CK and myoglobin compared to healthy controls (29). These elevated CK levels may hinder physiological adaptations to exercise, potentially accelerating disease progression and increasing the risk of end-organ damage linked to excessive CK release, such as rhabdomyolysis. As it has been shown that dystrophin is responsive to eccentric muscle work and that molecular adaptations to heavy exercise differ among individuals (22), sports with a high component of eccentric muscle work may not be suitable for MDCs.

In our two cases, repeated elevations in CK and troponin levels were detected and cannot be attributed to strenuous exercise alone. Troponin elevation in athletes can occur following exercise training, while the amount of elevation depends on both the intensity and duration (30). A recent study of 219 elite endurance athletes showed that an elevated troponin I level is a common finding in response to intense training, whereas troponin T elevation did not seem to be directly linked to physical exertion. Thus, troponin T, although not cardio-specific, may be better suited to rule out cardiac damage in athletes (31). However, there is evidence that troponin T is non-specific in skeletal muscle disorders and does not allow the differentiation between skeletal and cardiac muscle involvement (32). Thus, cardiac biomarkers in athletes with muscular disease may be non-specific and need to be interpreted in the context of kinetics, exercise intensity, and volume (30, 31, 33, 34).

Apart from the assessment of cardiac biomarkers, our two cases demonstrated that repetitive cardiac imaging plays a crucial role in the preparticipation screening and risk stratification of athletes. The decision regarding the eligibility of patients with cardiomyopathies to continue exercise is often co-determined by CMR imaging. This, however, is sometimes difficult, as LGE is often found in asymptomatic athletes without peripheral muscle pathology. Therefore, positive LGE in some MDC patients cannot be exclusively linked to myocardial involvement. However, LGE can, in general, be seen as a pathological sign, even in otherwise healthy athletes, of the potential existence of underlying genetic disease. LGE can have an impact on decision-making for athletes, as non-ischemic scars have been associated with an increased burden of malignant arrhythmias (35), while LGE in MDCs may facilitate the development of heart failure at a considerably young age (3). However, no reports exist on whether maintenance of high-intensity endurance exercise further exacerbates this risk in MDCs. Focal LGE at the right ventricular hinge point is a common finding in athletes and is not associated with malignant arrhythmias (36, 37). In a study on 72 recreational endurance athletes (mean age of 53 years) and 20 physically active individuals (mean age of 56 years), LGE was demonstrated in 33% and 20%, respectively, while there was no difference in extracellular volume (ECV) between the groups. In 23% of all the cases, LGE was located within the hinge region (36). In 93 highly trained endurance athletes (>12 h training/week in the previous 5 years; mean age of 36 years; 53% male), LGE was more prominent in the right ventricular insertion points compared to 72 age- and gender-matched controls (37.6% vs. 2.8%). In those athletes with LGE, T1 mapping revealed higher ECV in remote areas compared to those without (27 ± 2.2% vs. 25.2 ± 2.1%) (37). Moreover, 74 marathon runners were studied using CMR imaging to analyze the pattern of fibrosis (*n* = 55 men, mean age of 44 years; 19 women, mean age of 36 years). The study found LGE in 11% of the male and 13% of the female runners, with more than 50% of the LGE being located within the hinge region (38). Apart from genetic predisposition and silent myocarditis, prolonged exercise-induced pressure overload through high-volume exercise training may lead to microinjuries, resulting in localized fibrosis of the right ventricular insertion points and the release of cardiac enzymes (19, 38). No CMR studies that include MDC athletes exist; thus, international registries should include athletes with rare diseases such as this.

We propose an approach that integrates the kinetics of CK and troponin (ideally by longitudinal observation of troponin I and T kinetics), morphological changes, Holter monitoring, and exercise stress testing (Graphical Abstract). However, the limited sensitivity of exercise testing to induce arrhythmias has to be acknowledged. We also highlight that clinical decisions in athletes need to consider that resting LVEF can be reduced in the absence of structural heart disease (39), and this warrants the inclusion of stress echocardiography and follow-up examination. The cases in this study also illustrate that although CMR imaging is necessary to exclude acute myocarditis, which would trigger a temporary exclusion from structured sports, LGE is non-specific and requires careful expert interpretation and follow-up. Thus, an approach that leads to an interdisciplinary discussion with athletes with complex diseases who desire to participate in elite and recreational sports is mandatory (Graphical Abstract). The long-term impact of high-performance (both long-duration and high-intensity) exercise on patients with muscular disease and its implications on their long-term prognosis have not been studied sufficiently (21, 22). Therefore, shared decision-making by an interdisciplinary team of experts in the field of sports medicine is warranted. This is supported by evidence that the molecular adaptations of dystrophin to exercise vary among individuals, with potential harm to the muscle in sports that predominantly involve eccentric work (22). In genetic carriers of cardiomyopathies, a preparticipation evaluation with 12-lead ECG, echocardiography, maximal stress testing, and (preferably 12-lead) Holter monitoring should be mandatory (40). It should be stressed that there may be an increased risk of sudden cardiac death in MDC athletes displaying LGE and non-sustained ventricular tachycardia, necessitating continued cardiological follow-up. Furthermore, referral for electrophysiological investigation in cases of non-sustained ventricular tachycardia and left ventricular LGE should be considered in genetic cardiomyopathies (41–43).

In addition, the role of genetics in risk stratification (i.e., genotype–phenotype correlation) remains unclear due to limited sample sizes. Reports of a higher symptom burden in MDCs with duplications rather than deletions should not influence clinical decision-making. The decision of whether a patient should undertake further sports participation at a high-performance level needs to be made carefully to avoid stigmatization of otherwise healthy young individuals, but also to reduce the risk of malignant arrhythmias in affected athletes and consider the potential risk of premature heart failure in such patients. Shared decision-making with the athlete is warranted to weigh the benefits and risks of different modes of exercise (21, 22, 35, 36). We emphasize that athletes with rare diseases, such as dystrophinopathies, but also electrical diseases (e.g., long QT syndrome), who are willing to perform regular training, should be included in international sports cardiology registries, and the results should be shared with the sports cardiology community.

## 7 Conclusion

We provide the cases of two MDC athletes with potential cardiac involvement that necessitated interdisciplinary discussions on treatment decisions and clearance for competitive sports. These discussions considered clinical, genetic, and functional findings and the kinetics of cardiac biomarkers. To gather more evidence for future decision-making, all athletes with rare diseases should be followed up in international registries.
